# Computed tomographic colonography: how many and how fast should radiologists report?

**DOI:** 10.1007/s00330-019-06175-y

**Published:** 2019-04-08

**Authors:** Anu E. Obaro, Andrew A. Plumb, Michael P. North, Steve Halligan, David N. Burling

**Affiliations:** 1 Centre for Medical Imaging, Podium Level 2, University College London Hospital, Euston Rd, London, NW1 2BU UK; 2 St Mark’s Academic Institute, St Mark’s Hospital, Harrow, UK

**Keywords:** Colorectal neoplasms, Computed tomographic colonography, Diagnostic errors, Workflow, Fatigue

## Abstract

**Objectives:**

To determine if polyp detection at computed tomographic colonography (CTC) is associated with (a) the number of CTC examinations interpreted per day and (b) the length of time spent scrutinising the scan.

**Methods:**

Retrospective observational study from two hospitals. We extracted Radiology Information System data for CTC examinations from Jan 2012 to Dec 2015. For each examination, we determined how many prior CTCs had been interpreted by the reporting radiologist on that day and how long radiologists spent on interpretation. For each radiologist, we calculated their referral rate (proportion deemed positive for 6 mm+ polyp/cancer), positive predictive value (PPV) and endoscopic/surgically proven polyp detection rate (PDR). We also calculated the mean time each radiologist spent interpreting normal studies (“negative interpretation time”). We used multilevel logistic regression to investigate the relationship between the number of scans reported each day, negative interpretation time and referral rate, PPV and PDR.

**Results:**

Five thousand one hundred ninety-one scans were interpreted by seven radiologists; 892 (17.2%) were reported as positive, and 534 (10.3%) had polyps confirmed. Both referral rate and PDR reduced as more CTCs were reported on a given day (*p* < 0.001), the odds reducing by 7% for each successive CTC interpreted. Radiologists reporting more slowly than their colleagues detected more polyps (*p* = 0.028), with each 16% increase in interpretation time associated with a 1% increase in PDR. PPV was unaffected.

**Conclusions:**

Reporting multiple CTCs on a given day and rapid CTC interpretation are associated with decreased polyp detection. Radiologists should be protected from requirements to report too many CTCs or too quickly.

**Key Points:**

*• CT colonography services should protect radiologists from a need to report too fast (> 20 min per case) or for too long (> 4 cases consecutively without a break).*

*• Professional bodies should consider introducing a target minimum interpretation time for CT colonography examinations as a quality marker.*

## Introduction

Year on year, medical imaging increases inexorably [1, 2], outstripping growth in radiology staffing [3]. Radiologists are therefore under increasing pressure to report more studies more rapidly, while maintaining accuracy. Yet these two demands conflict fundamentally; faster reporting may compromise image scrutiny, leading to increased error. For simpler examinations consisting of a single or very few images (e.g. conventional radiographs), rapid interpretation may be possible with relatively little deterioration in diagnostic performance [4]. Conversely, for complex studies with multiple images (e.g. CT scanning), greater interpretation speed is commonly deleterious [5].

Computed tomographic colonography (CTC) is both complex and time-consuming to interpret. Moreover, it is fatiguing, as the interpretive task (of flying or scrolling through the colon) is repetitive, and the majority of examinations are negative for the primary target condition (colorectal cancer or large polyps) [6–9], a phenomenon that is known to reduce vigilance [10]. Anecdotally, radiologists often admit that they find it tiresome to report more than a handful of CTC examinations in a given reporting session, and that their concentration often wavers if they attempt to do so. It is therefore tempting to interpret CTC rapidly, particularly for the final few examinations in a given reporting session. However, this may well reduce detection rates; in a laboratory environment, more rapid fly-through at endoluminal CTC reduces both the proportion of colonic mucosa viewed by the radiologist and the polyp detection rate [11]. For colonoscopy, endoscopists with shorter withdrawal times (i.e. providing less time to inspect the colon) have lower adenoma detection rates (ADR) [12–14] and higher interval cancer rates [14]. Moreover, ADR tends to drop towards the end of the day and even towards the end of an individual colonoscopy list [15, 16], implying a “fatigue effect” when performing multiple examinations consecutively. Whether the same is true for CTC is unknown.

We therefore aimed to determine if polyp detection rates (PDR) and positive predictive value (PPV) at CTC are associated with (a) the number of CTC examinations interpreted by a radiologist on any given day (i.e. a fatigue effect) and (b) the length of time radiologists spend on interpretation (as a proxy for completeness of image scrutiny).

## Materials and methods

### Data collected

This retrospective study used routinely collected data and was approved as a service evaluation by the relevant departments. We collected data from the Radiology Information Systems for all CTC examinations reported by seven gastrointestinal radiologists at two centres, spanning the period January 2013 to December 2015 (centre 1) and January 2012 to December 2015 (centre 2). We only included radiologists who had interpreted more than 200 CTC examinations during this period, to ensure percentages could be calculated with sufficiently narrow 95% confidence intervals to be meaningful. All radiologists had pre-existing CTC expertise; each was a gastrointestinal radiologist, had undergone specific training and had interpreted > 500 examinations. Both centres employed a similar CTC protocol during this period, employing normal-dose post-contrast supine and low-dose prone scans after combined purgation and faecal tagging, intravenous spasmolytics (hyoscine butylbromide), and automated carbon dioxide insufflation. For each radiologist, we extracted (a) the date and time of report verification for all examinations they had reported during this period and (b) the full text of any CTC examinations reported during that period. Subsequently, we inspected the CTC reports to determine if the radiologist had, or had not, reported a 6-mm+ polyp or colorectal cancer. We used each hospital’s patient record system to ascertain whether or not patients with a positive CTC underwent confirmatory testing (i.e. endoscopy or surgery) and, if so, whether the CTC finding was a true positive or false positive. We regarded the presence of any endoscopically or surgically proven polyp or cancer as a true-positive CTC finding, regardless of location or final histology (i.e. a per-patient match). For each radiologist, we estimated their potential “referral rate” (defined as the proportion of CTC examinations in which they reported a 6-mm+ polyp or cancer, i.e. that might be expected to precipitate a referral for colonoscopy), their positive predictive value (PPV; defined as the percentage of cases in which a polyp or cancer was ultimately found if confirmatory testing was done) and their polyp detection rate (PDR; defined as the proportion of cases in which a polyp or cancer was ultimately confirmed, relative to the total number of cases interpreted). For all these proportions (expressed as percentages), 95% confidence intervals were estimated using the Wilson method [17].

### Estimation of time taken for CTC interpretation

Using the extracted dates and times of each radiologist’s complete reporting record for this period, for any given CTC examination, we recorded whether it was the first, second, third (and so on) CTC study reported by the radiologist on that particular day. We also estimated the length of time taken to interpret each CTC examination by deducting the time of report verification for a given CTC study from the time at which the immediately preceding report was verified. For example, if a radiologist verified a chest radiograph at 9:00 a.m., and their next report verification was a CTC examination at 9:30 a.m., we assumed that the radiologist had spent 30 min interpreting the CTC. If a CTC examination was the first report verified on a particular day, it was retained for the purposes of estimating each radiologist’s referral rate, PPV and PDR, but not included when estimating reporting time (since there was no immediately preceding examination to calculate interpretation time). Since CTC examinations that are positive for polyps or cancers take longer to interpret than those that are negative, for each radiologist we calculated their negative interpretation time (by analogy with the colonoscopic negative withdrawal time), by taking the mean of the estimated CTC interpretation time for cases in which no polyp was reported. This better reflects image scrutiny alone (i.e. a normal case) rather than combining both scrutiny and interpretation (e.g. detection followed by characterisation and measurement). To allow for interruptions and batch verification of multiple reports dictated at an earlier time, we set plausible limits on CTC interpretation times; any CTC that appeared to take less than 5 or more than 60 min were assumed to have been pre-reported (and therefore re-checked or verified), or reported after an interruption respectively, and were excluded. Both sites had both 2D and 3D interpretation software available, although at one site this was a thin client launched from the PACS, whereas at the other it was a stand-alone workstation. Computer-aided detection (CAD) was not used routinely at either site. Since the two institutions investigated had different CTC interpretation workflows (e.g. availability of CTC workstations), voice recognition systems and RIS software, we presented negative interpretation time as a proportion relative to their colleagues at the same centre, by dividing by the centre mean.

### Analysis

To assess the effect of interpreting multiple CTC examinations on a given day, we calculated the referral rate and polyp detection rate grouped by the sequence in which the CTC was reported (i.e. first CTC reported that day, second, etc.). To estimate effect size and statistical significance, we used multilevel logistic regression (three levels: CTC, radiologist, centre), with the presence of a polyp as the binary outcome variable and sequence in which the CTC had been performed as the main explanatory variable. To assess the effect of interpretation time on polyp detection, we compared the negative interpretation time for each radiologist with their referral rate, PDR and PPV. We assessed statistical significance using linear regression, with referral rate, PDR and PPV as the outcome variables and negative interpretation time as the explanatory variable. All analysis was performed using R version 3.5.1 for Mac [18].

## Results

### Radiologist referral rate, PDR and PPV

Overall, 5191 CTCs were reported by the 7 radiologists. The individual radiologist referral rate, PDR and PPV are shown in Table 1. There was a moderate spread in the referral rate and PDR, ranging from 13.1 to 27.5% for the referral rate and 7.8 to 16.3% for the PDR. PPV was grouped more tightly, ranging from 83.3 to 96.1%. The radiologist with the highest PDR had the lowest PPV, and the radiologist with the highest PPV had the second lowest PDR, but overall there was no consistent relationship between radiologist-level PPV and PDR (weak negative correlation, Pearson *r* = − 0.51, *p* = 0.25). Overall, both the referral rate and PDR were higher at centre 1 than at centre 2 (referral rate, 19.7% vs 16.1%, *p* = 0.0019; PDR, 12.2% vs 9.5%, *p* = 0.0039), but PPV was lower (85.3% vs 93.5%, *p* = 0.0018).

**Table 1:** Number of CTC studies, referral rate, confirmatory testing, positive predictive value (PPV) and polyp detection rate (PDR), split by radiologist and study centre. Numbers in brackets indicate 95% confidence intervals

Radiologist	Number of CTC studies interpreted	Number interpreted as positive	Referral rate % (95% CI)	Number undergoing confirmatory testing	Number with polyp or cancer confirmed	PPV % (95% CI)	PDR % (95% CI)
1	338	93	27.5 (23.0 to 32.4)	66	55	83.3 (72.6 to 90.4)	16.3 (12.7 to 20.6)
2	268	51	19.0 (14.8 to 24.2)	43	37	86.0 (72.7 to 93.4)	13.8 (10.2 to 18.4)
3	964	165	17.1 (14.9 to 19.6)	115	99	86.0 (78.6 to 91.2)	10.3 (8.5 to 12.4)
Centre 1 totals	1570	309	19.7 (17.8 to 21.7)	224	191	85.3 (80.0 to 89.3)	12.2 (10.6 to 13.9)
4	461	106	23.0 (19.4 to 27.0)	74	67	90.5 (81.7 to 95.3)	14.5 (11.6 to 18.0)
5	694	91	13.1 (10.8 to 15.9)	60	54	90.0 (79.9 to 95.3)	7.8 (6.0 to 10.0)
6	410	81	19.8 (16.2 to 23.9)	51	49	96.1 (86.8 to 98.9)	12.0 (9.2 to 15.5)
7	2056	305	14.8 (13.5 to 16.6)	182	173	95.1 (90.9 to 97.4)	8.4 (7.4 to 9.8)
Centre 2 totals	3621	583	16.1 (14.9 to 17.3)	367	343	93.4 (90.5 to 95.6)	9.5 (8.6 to 10.5)
Grand total	5191	892	17.2 (16.2 to 18.2)	591	534	90.4 (87.7 to 92.5)	10.3 (9.5 to 11.1)

### Diagnostic yield by number of CTC examinations reported on a given day

**Fig. 1:**
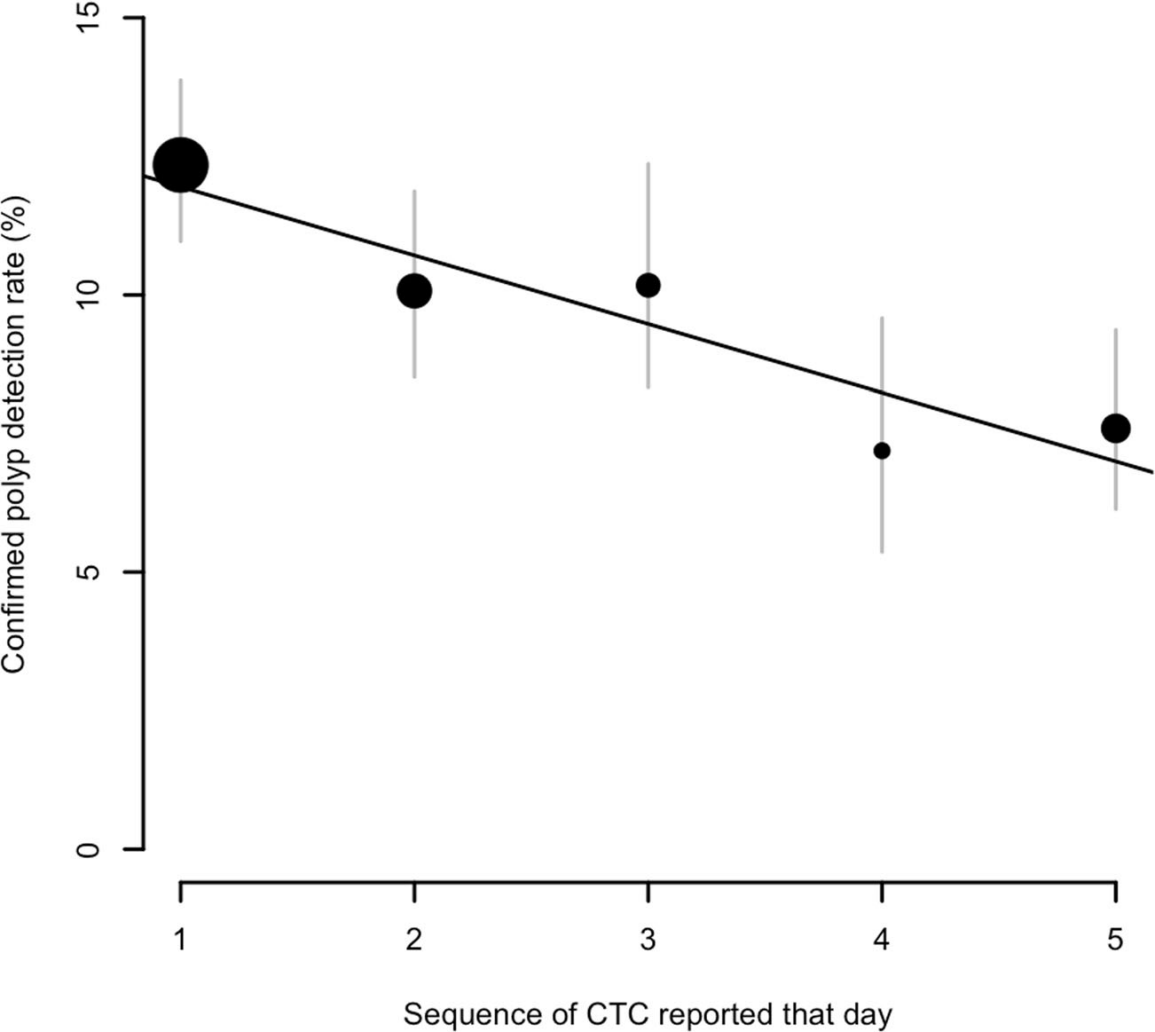
Confirmed polyp detection rate for each CTC reported in a given day. The area of the marker is proportional to the number of scans in each category; grey bars indicate 95% confidence intervals. The line corresponds to a fitted linear trend

The rate of positive CTC examinations declined with increasing numbers interpreted on a particular day (Fig. 1). For the first CTC study reported, 21.7% (95% CI, 19.9 to 23.6%) were believed positive for 6-mm+ polyps or cancer by the radiologists, with 12.3% (95% CI, 11.0 to 13.9%) ultimately having polyps or cancer confirmed. By the time of the fifth (or greater) CTC interpretation, only 13.7% (95% CI, 11.7 to 15.9%) were interpreted as abnormal, with a mean PDR of 7.6% (95% CI, 6.1 to 9.4%) for such studies. Therefore, an approximately 40% decline in polyp detection occurred during the day when multiple CTC studies were reported. This was highly statistically significant, with an odds ratio of 0.93 (95% CI, 0.88 to 0.97; *p* < 0.001) for referral rate (i.e. abnormality identified at CTC) and an odds ratio of 0.93 (95% CI, 0.90 to 0.97; *p* < 0.001) for polyp detection (i.e. confirmed at endoscopy or surgery). Therefore, for each successive CTC study reported on a given day, the odds of both identifying and confirming a polyp at CTC dropped by 7%. There was no consistent effect of reporting multiple CTC examinations on PPV, which remained consistent at around 90% regardless of examination sequence (*p* = 0.11).

### Negative interpretation time and radiologist detection rates

**Fig. 2:**
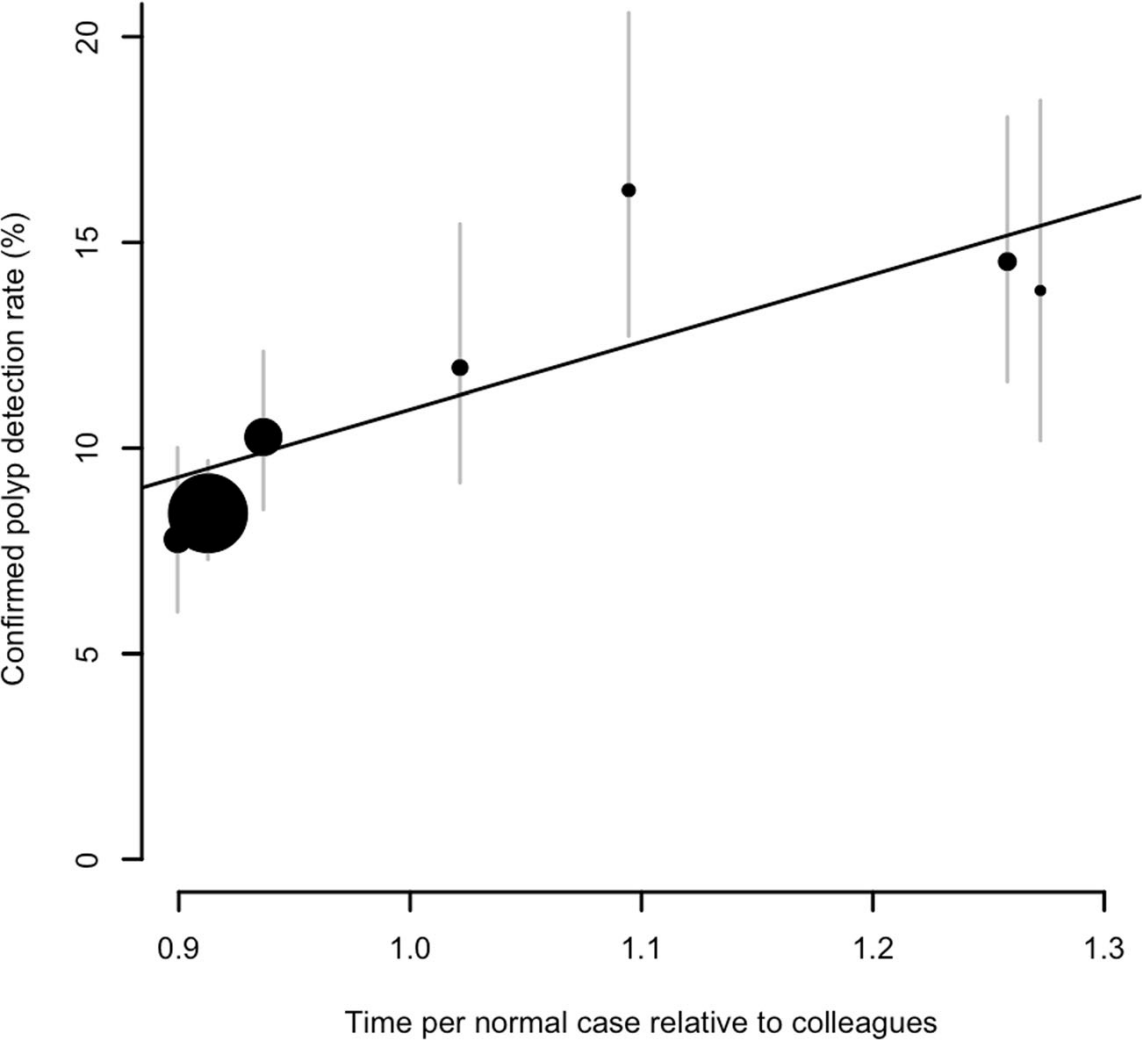
Confirmed polyp detection rate against the average length of time spent by a radiologist on interpreting a negative case (i.e. negative interpretation time). Each marker corresponds to a single radiologist; the area of the marker is proportional to the number of scans reported by that radiologist, with grey bars indicating 95% confidence intervals. The line corresponds to a fitted linear trend

Three hundred twenty-nine CTCs were reported as the first examination on a given day, and so the interpretation time for these could not be estimated. For the remaining 4862 studies, the mean time taken to interpret a negative CTC examination was 30.5 min (centre 1, 17.4 min; centre 2, 34.6 min). Overall, there was a weak positive association between negative reporting time and PDR; radiologists who spent longer interpreting cases that they ultimately called normal detected more polyps than those who reported more quickly (Fig. 2). This effect was small but statistically significant (*p* = 0.028), with the regression model suggesting that each 16% increase in interpretation time was associated with a 1% increase in detection rate. There was no clear relationship between negative interpretation time and PPV (*p* = 0.478).

### Number of CTC examinations interpreted and time spent on image scrutiny

As the number of CTC studies reported on a given day increased, the mean time spent interpreting each study reduced at centre 1 (reducing from a mean of 19.9 min for scan 1 to a mean of 16.4 min by the time five or more scans had been interpreted), a statistically significant reduction (*p* = 0.0012). Conversely, the negative interpretation time remained constant at centre 2, irrespective of how many scans had been reported that day (*p* = 0.59).

## Discussion

CTC interpretation is time-consuming and fatiguing. We found that as radiologists interpreted more CTC examinations on a given day, their detection rate dropped by roughly 40% after five or more studies had been reported. Moreover, radiologists who spent longer interpreting cases that they ultimately called negative had higher detection rates than their colleagues who interpreted more quickly, with no corresponding detriment to their positive predictive value. These data strongly suggest that radiologists reporting CTC must be protected from pressures to report too quickly, or for too long—or missed pathology will be the consequence.

Although, in most cases, the primary goal of CTC is to confirm or refute colorectal cancer (or an alternative cause for patient symptoms, such as diverticulosis), it also represents an opportunity to reduce future colorectal cancer incidence by detection and subsequent removal of precursor polyps (i.e. adenomas and certain serrated lesions). Accordingly, radiologists interpreting CTC must be vigilant not only for large masses that may underpin symptoms, but also for smaller polyps; otherwise, patients may return in the future (usually many years later) with a post-investigation colorectal cancer (PICRC) [19]. Indeed, the majority of PICRCs occurring after CTC are visible in retrospect, either as a mass lesion that was overlooked or as a polyp that subsequently became malignant [20]. Such errors will be impossible to prevent entirely, but systems and methods that diminish this clinical and medicolegal risk would improve patient care substantially. Our findings suggest that relatively simple changes to radiologist workflow might be valuable; avoiding fatigue by reducing the number of CTC studies reported consecutively and introducing a minimum “negative interpretation time” before a scan are deemed normal. This is highly plausible, because eye-tracking experiments show that over-rapid endoluminal fly-through reduces the amount of colonic surface that a radiologist can bring into their central vision [21]—slowing down would mitigate this risk. A minimum interpretation time of 20 min per case would seem reasonable, since this was the average time taken for the first scan interpreted each day at the quicker of the two centres, with 30 min per negative case being a desirable (and achievable) standard.

Of note, for the most directly comparable test to CTC, namely colonoscopy, the importance of prolonging inspection of the colonic mucosa to maximise detection has been recognised for many years [12]. Gastroenterologists who spent less than 6 min withdrawing the colonoscope had detection rates that were less than half that of their colleagues spending longer [12]. More recently, data from the English Bowel Cancer Screening Programme (in which endoscopists are already highly trained and accredited) show that extending the examination towards 10 min yields further benefits in detection rate [13]. Moreover, this translates to clinical outcomes; colonoscopists with longer withdrawal times have lower post-colonoscopy colorectal cancer (PCCRC; similar to “interval cancers” in a screening programme) rates than those who withdraw the scope (too) rapidly [14]. Negative withdrawal time (i.e. calculated only for cases where no polyps are found) is now recognised as a key performance indicator (KPI) for the quality of many colonoscopy services, including in the UK [22], Europe [23] and the USA [24].

The concept of slowing down to improve accuracy is not new, nor is it specific to CTC. Requirements to report large numbers of examinations rapidly (to reduce wait times and reporting backlogs) must be balanced against the risks of making errors. If scans are acquired but languish on the PACS, remaining unreported, this is a worse situation than them being reported, even suboptimally. This clinical risk has been highlighted in England by the Care Quality Commission (CQC) [25]. On the other hand, patients will rightly not accept that their cancer or polyp was missed due to time pressures and underfunding. It is highly iniquitous and counter-intuitive that a patient may have colonoscopy, where they receive the undivided attention of an accredited endoscopist who will examine their colon for a minimum length of time (i.e. the negative withdrawal time), or—based on local pathways or the whim of a referring doctor—instead undergo CTC where the radiologist may be interrupted repeatedly and without any minimum standard for the duration of interpretation. Such infrastructural and process shortcomings highlight the need for robust minimum standards that protect both patients and radiologists in the face of increasing demand.

We also found that reporting multiple CTC examinations in sequence was associated with progressive deterioration in detection, suggesting a “fatigue effect”. This phenomenon has been described in many other areas, including colonoscopy. The adenoma detection rate (ADR) falls as colonoscopy lists progress [15, 16], and is typically higher in the morning than evening. However, this finding is not universal, and some studies have found the effect either weak [26] or absent entirely [27]. Nonetheless, anecdotally, many radiologists become fatigued after reporting several CTC examinations consecutively, and avoid doing so where possible. Given our findings, it may be prudent to avoid reporting large numbers (four or more) of CTC without a break. A 4-h session of approximately eight CTC studies reported in two blocks with a half-hour break would seem an appropriate guideline, since it permits both the minimum negative interpretation time and no more than four cases criteria to be met.

This study has several limitations. Firstly, we investigated just two tertiary care centres, and only seven radiologists, which may not represent wider practice. Secondly, the data are retrospective and observational, and therefore it is not possible to exclude bias. For example, scans interpreted earlier in a sequence may have been highlighted to the radiologist for prioritisation (for example, marked as “urgent” on the RIS), although it was the practice at both institutions to report in date order. Even so, if we ignore fatigue, it is difficult to explain why the effect of scan sequence was consistent across two centres with different workflows and for as many as five successive scans. Thirdly, we were forced to make some assumptions when estimating radiologist negative interpretation time, specifically calculating the time spent interpreting a CTC study by using the time at which the report was verified and relating this to the immediate prior report, and excluding some reports with implausibly long or short interpretation times. The reporting time also includes time spent scrutinising the image for extracolonic findings, which may partly explain the relatively large difference in average interpretation time between the two centres. We mitigated against this by using each radiologist’s negative interpretation time normalised to the centre average, but this may have altered the size of the effect that we observed.

In summary, in a retrospective observational study from two NHS hospitals, we found that the proportion of positive CTC examinations and polyp detection rates reduced as radiologists reported multiple examinations, suggesting a “fatigue effect”, and radiologists with longer interpretation times had higher polyp detection rates with no corresponding reduction in positive predictive value. CTC services should protect their radiologists and patients by removing the need to report too fast or for too long.

## Abbreviations

ADR: Adenoma detection rate
CRC: Colorectal cancer
CTC: Computed tomographic colonography
PCCRC: Post-colonoscopy colorectal cancer
PDR: Polyp detection rate
PPV: Positive predictive value
RIS: Radiology information system
